# Review of Recent Publications in Medical Zoology

**Published:** 1892-02

**Authors:** C. W. Stiles

**Affiliations:** Ph. D. (Washington, D. C.)


### REVIEW OF RECENT PUBLICATIONS IN MEDICAL ZOOLOGY.

C. W. Stiles, Ph. D. (Washington, D. C.).

### PROTOZOA.

A number of articles have recently appeared on pathogenic protozoa (Danilewsky, Grassi and Feletti, Pfeiffer, etc.), discussing the morphological interpretation of various forms, especially of the malarial parasites. Metschnikoff and others include the malarial parasite in the Coccidia, while Kruse and others contend that this parasite is a Gregarina, since the adult stage is found free in the blood.

Grassi and Feletti (Malariaparasiten in den Vögeln, Centralbl. f. Bakt. u. Parasit. 1891, IX.) take still another view. They admit two genera among the malaria parasites : *Hæmamœba* with three species *H. præcox*, as cause of quotidian fever, *H. vivax*, as cause of tertiana, and *H. malarial* as cause of quartana, while *Laverania* causes an irregular fever. *Hæmamœba* is supposed to correspond to *Amœba guttata*, and *Laverania* to *Amœba radiata*. The parasites thus being brought into the cyclus of Amœba, would, according to these authors, belong to the *Rhizopods* instead of the *Sporozoa*, a view which, in face of comparative parasitology, we are not ready to accept.

L. Pfeiffer has just published a second edition of his pamphlet on pathogenic protozoa ; in this work, which is much larger than the first edition, Pfeiffer lays great stress upon the recent discovery by his nephew, R. Pfeiffer, of the so-called *Gymnospores* (naked spores) in the Coccidia. Heretofore, every Coccidium cyst (found in man, cattle, horses, dogs, cats, and especially frequent in the liver of rabbits,) has been looked upon as the growth from a spore, which entered the animal through its food or drink. R. Pfeiffer has, however, shown that *gymnospores* may germinate from the young parasites, and that these naked (gymno-) spores serve to spread the infection inside the infested host, while, as was already known, the so-called final spore (Danerspore of the Germans), serves to spread the infection among other animals. Kruse, of Naples, has arrived at the same results that Pfeiffer has, although entirely independently. According to Kruse the gymnospores can be formed at most any time during the development of the parasite. These new results make the Coccidia more dangerous than was formerly supposed, since a slight infection may, by means of the gymnospores, become a heavy infection.

Many authors consider a species of Coccidium as the cause of carcinome, but Prof. Ribbert, of Bonn (Ueber Einschlüsse im Epithel der Carcinome; Deutsche Med. Wochenschrift, 1891, p. 1179–1183), who has studied the matter very carefully, comes to the conclusion that the peculiar figures noticed in carcinome, and described by several authors as parasitic *Coccidia*, are "nothing but changed and degenerated epithelial cells or their nuclei."

Danilewsky stated recently (Central-bl. f. Bakt. u. Parasit. 1891. IX. 1.) that he had found parasites belonging to the group "Mikrosporidia," in the muscles of frogs, lizards, and turtles. A portion of muscle from a turtle, said to have come from D.'s original material, was handed me for examination. In the portion I examined no such parasites were found; I found, however, small vacant spaces between the muscle fibres, and evidently caused by their contraction. It seems hardly possible that so careful an investigator as Prof. Danilewsky could have mistaken these air-bubbles for microsporidia, parasites found chiefly in insects and small crustacea (for instance, pebrine of the silkworm), yet in this piece of meat there were certainly no parasites present.

CESTODES.

Dr. Moore recently presented a paper before the Washington (D. C.) Biological Society, describing a case of *Hydatid Echinococcus* in pig. In the discussion which followed Stiles spoke of two other unpublished cases of the occurrence of this parasite in American animals, and one case of its occurrence in man; this last case, where death resulted from the presence of the parasite, has since been published by Dr. Van Cott, of Brooklyn, N. Y.

Prof. Edwin Linton, of Washington, Penn., has recently given us several valuable contributions to parasitology. His results, however, are of more importance to the zoologist than to the veterinarian, since all the parasites mentioned come from fish. In one paper (Bulletin of the U. S. Fish Commission, IX., p. 337) he shows that *Dibothrium cordiceps*, a larval form found in the trout of the Yellowstone Park, develops into an adult tapeworm in the intestines of the white pelican (*Pelecanus erythrorhynchus*). Prof. Linton's paper "Notes on Entozoa of Marine Fishes of New England, II. (Bul. cit., 1890), contains descriptions of forty-two parasites, many of which are new.

To Prof. Blanchard we owe another paper on tapeworms (Notices helminthologiques, Mém. d. l. Soc. Zool. d. France, 1891, p. 420–480). The tapeworms, with armed suckers, he includes in three genera :

1. Echinocotyle gen. nov. with *E. Rosseteri* R. Bl. '91, as type. This is a small (3 mm.) tapeworm of the duck. The rostellum is armed with ten hooks, and each sucker with about ninety-seven smaller hooks, arranged in a circle around the border of the sucker, and in a central longitudinal row. The larval stage is found in a small crustacea (*Cypris cinerea*). This parasite has, as yet, been found only ien Bngal ducks.

2. Davainea.—R. Bl. and Railliet, '91 gen. nov. The rostellum armed with a double crown of hooks, suckers armed with several rows of hooks. A. Genital pores alternate. 1. *Davainea proglottina*, Dav., 1860, found in chickens. 2. *D. echinobothrida*, Mégnin, 1881, found in chickens, pheasants and pigeons. . . . 4. *D. cesticillus*, Molin, 1861, chickens and pheasants. According to Grassi and Rovelli, the secondary host is a beetle or butterfly. B. Sexual pores uni-lateral . . . 9. *D. tetragona*, Molin, 1861, chickens. According to Piana, the larvæ are found in snails (*Helix carthusianella*, *H. maculosa*). 10. *D. columbae*, Zeder, 1800, pigeons. . . . 12. *D. Friedbergeri* v. Linstow, 1878, in pheasants; frequently fatal! . . . 14. *D. madagascariensis*, Davaine, 1869, (Tænia madagascariensis) in man.

3. Ophryocotyle.—Friis, 1869. The rostrum is wanting, but head is provided with several infundibula, the borders of which are armed with numerous hooks. Suckers round and armed with several transverse rows of hooks. Three species present in various birds.

Professor Blanchard unites in the sub-family *Anoplocephalinæ* all the unarmed tapeworms of the Tænia family, occurring in herbivorous animals and having certain characters in common, one of which is the pyriform apparatus of the ovum. Three genera are included in this sub-family. The following are the forms of interest to veterinarians; for details see original article: MONIEZIA.—1. *M. alba* (Tænia alba) of sheep; 2. *M. Bendeni*, in sheep; 3. *M. denticulata*, sheep and cattle; 4. *M. expansa*, sheep and cattle; 9. *M. Neumanni*, Moniez, '91, sheep; 10. *M. nullicollis*, Moniez, '91, sheep. ANOPLOCEPHALA.—5. *A. mamillana*, horse; *A. perfoliata*, horse; *A. plicata*, horse. In these species the segments are broader than long. The genus Moniezia is characterized by the presence of two sexual pores to each segment, while the genus *Anoplocephala* has sexual pores on only one side of the body. From the specific names given above, it will be recognized that nearly all the forms were originally described as belongiug to the genus *Tænia, as Tænia expansa*, etc.

The other parasites mentioned in Blanchard's paper are of more scientific than economic interest.

In another publication (Histoire Zoologique et Médicale des Téniadés du Genre Hymenolepis, Paris, 1891), Blanchard gives a revision of those members of the family *Tæniadæ* which are included in the genus *Hymenolepis*, characterized by the presence of but three testes in each segment and by the genital pores piercing the left border (for further particulars of the diagnosis see the original). Of the species included in this genus, two are of particular interest to the physician, since they occur in man. 1. *Hymenolepis nana* (*Tænia nana*) is, according to Professor Grassi, identical with *H. murina* of the rat, but according to Blanchard and other zoologists, it forms a distinct species. The question as to the identity of these two species is extremely important, since Grassi has shown that *H. murina* has an indirect development WITHOUT change of host; that is, the embryo develops in the villosities of the intestine into the larval stage, which then falls into the lumen and becomes an adult worm. This is the only species in the Tænia family which has been proven to to have a development without intervention of a secondary host. If the two species mentioned are identical, then we must assume a similar development for this parasite in man. Although *H. nana* is the smallest tapeworm found in man, measuring but 10-25 mm. in length, it is by no means a harmless parasite. *Symptoms:* Pains in the abdomen, gastro-enteric disorders, sometimes lasting several years, diarrhea, appetite good to insatiable; general feebleness, cachectic aspect; dyspnoea. Nervous complications may arise; trouble in vision or speech; epileptic fits, even brain troubles, meningitis, melancholy. *Diagnosis:* Microscopic examination of faeces to find the eggs. In Italy, where this parasite has been attracting a good deal of attention of late years, the following treatment is recommended: Six grammes of extract of male fern in half a glass of gum water (Grassi); or first day (evening), two doses of santonine (20 centigrammes); second day (morning), castor oil; third day (morning), 3 grammes extract fern and 3 centigrammes of calomel (sonsino).

The second species of interest in this treatise is *H. diminuta*, known to physicians as *Tænia flavopunctata;* it is 20-60 cm. long, and is generally found in certain rodents, such as the house-rat, but has also been found four times in man. The larval stage is developed in insects, such as *Asopia farinalis* (a butterfly), *Akis spinosa* (a beetle), etc.

NEMATODES.

Willach describes a new and dangerous roundworm, found in nodules of the colon of certain apes (*Macacus cynomolgus*) (Arch. f. w. u. pr. Thierheilkunde, 1891, p. 340–346). The new parasite, which receives the name *Sclerostoma apiostomium*, caused colitis hæmorrhagico-diphtheritica, resulting fatally in two out of three cases examined.

C. W. Stiles (Sur la Deut. des Embryons d'Ascaris, Bull, d. l. Soc. Zool. d. France, 1891, p. 162), describes the so-called boring tooth of *Ascaris lumbricoides* (of man, pigs and sheep) as three lips corresponding to the lips of the adult worm.

ACANTOCEPHALA.

Two very fine monographs of the genus Echinorhynchus have recently appeared in Germany, one by Kaiser, the other by Hamann, but as they treat the subject almost entirely from a zoological standpoint, we can do no more than mention them here. As I already stated in my article an Echinorhynchus (December number of this journal), Kaiser infected the German gold-beetle (*Cetonia aurata*) with eggs of Echinarhynchus gigas of the hog, and looks upon that insect as the regular secondary host for this parasite in Germany.

ARACHNOIDEA.

C. W. Stiles (Bau und Entwicklungsgeschichte von Pentastomum proboscideum und Pentastomum subcylindricum, Zeitschrift für wiss. Zool., 1891, p. 85–157, and Sur la Biologie des

Linguatules—Comp'l. rend. d. l. Soc. d. Biologie, 1891). The only portions of the above papers which are of direct interest to the veterinarians are those treating of the migrations of the various, species of Pentastomum. All the theories advanced by various authorities in regard to the migration of these curious worm-like parasites are contained in the following table.

The embryos, which are surrounded by three shells, are expelled from the adult female parasite and escape from the host :

I. Through the nostrils (P. Tænioides of the dog), after R. Leuckart.

II. Through the anus or nostrils (P. proboscideum of snakes), after Stiles.

These embryos are scattered on plants or fall into the water, and are swallowed by a secondary host (man, rabbits, sheep, horses, cattle in case of P. tænioides (Leuckart and others) the mouse and various other small mammals in the case of P. proboscideum (Stiles). On arriving in the stomach, the eggshells are digested and the embryo, which is thus freed, bores its way into the various organs (liver, lungs, etc.), and there develop into the encysted larval stage.

***A***. *As a rule*, these larvæ arrive in the definite host, when the latter devours the secondary host (Leuckart, Stiles).

(*a*) If the larvæ have already escaped from their cyst, they pass directly through the nostrils [?] into the nose, or are taken into the mouth and pass from here into the nasal cavities (P. tænioides in dogs), according to Lenckart.

(*b*) When the larvæ are still encysted on arriving in the final host, they pass into the stomach, where the cyst is digested ; then they penetrate the intestinal walls and wander through the body by way of the lungs and trachea to the nose (P. tænioides, after Gerlach), (P. proboscideum, according to Stiles). In serpents, which always eat their prey without chewing it, the larvæ must, of course, always pass into the stomach before reaching their final seat in the air passages. It is *not impossible* that some larvæ crawl from the stomach through the œsophagus to the nose.

***B***. Three *exceptions* are possible to the above rule (Stiles).

(*c*) When the larvæ arrive in the stomach of the final host, they sometimes encyst themselves in the various organs before arriving at their definite habitat (Stiles). In this way some cases are explained, in which larvæ have encysted in certain carnivorous animals : P. moniliforme, encysted in the lung tissue of the Python snake (Jacquart) ; P. proboscideum in the lung tissue of the Boa constrictor, and, upon infection, free in the body cavity of other snakes (Stiles) ; *perhaps*, also, for P. tænioides in the cat, and for some of the cases of encysted P. tænioides in man. If this latter supposition is correct, man would be open to infection from two sources, *i. e.*, from the dogs, in becoming infected with the embryos, and from eating rabbits infected with larvæ.

(*d*) If the larvæ bore through the intestines (Babes) or bronchial tubes (Gerlach) of the secondary host, they might be expelled through the mouth or anus, and arrive in the definite host by means of the food. *Babes considers this the regular mode of migration for the Pentastomes of cattle, while Stiles looks upon it as a possible, but rare, exception to the rule.*

(*e*) If the encysted larvæ break their cyst and fall into the abdominal cavity of the secondary host, they might bore their way to the nasal cavity and develop there into the adult form, thus offering a case of development without change of host (Chatin, for certain species, Laudan, case of a German soldier). The possibility of this exception is admitted by R. Blanchard and Stiles. All cases in which adult Pentastomes are found in the nasal cavities of herbivarous animals are to be explained, either in this way, or by an exceptional feeding upon meat, or by accidental introduction of the larva with the food, etc.

